# Supplementary figures and images for: The interferon-inducible GTPase MxB promotes capsid disassembly and genome release of herpesviruses
Source: eLife. 2022 Apr 27;11:e76804. doi: 10.7554/eLife.76804 (PMC9150894; doi:10.7554/eLife.76804)

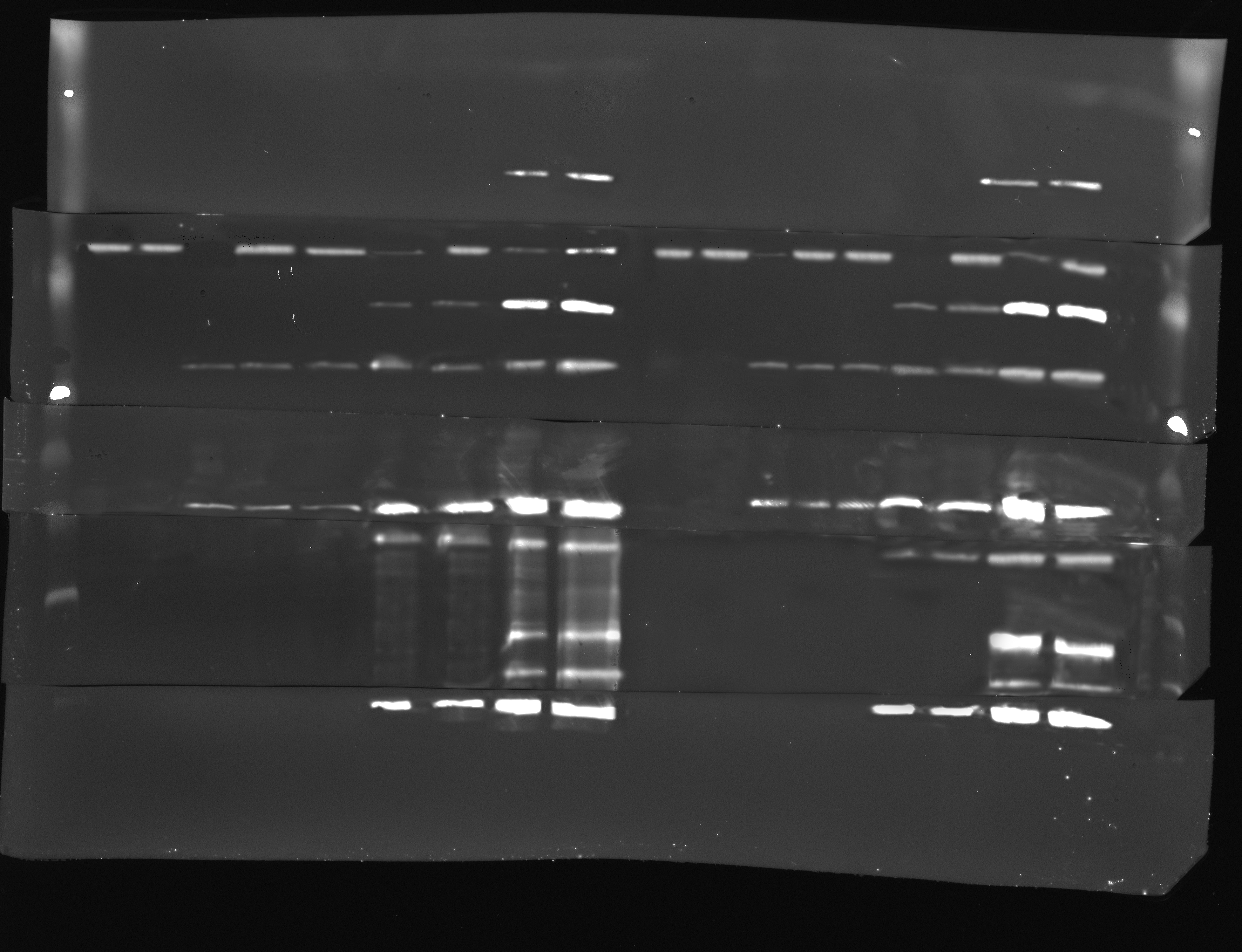

Supplement: Figure 2—figure supplement 1—source data 1. [file elife-76804-fig2-figsupp1-data1.zip › Figure 2-figure supplement 1-source data 1-exposure adjusted.TIF]

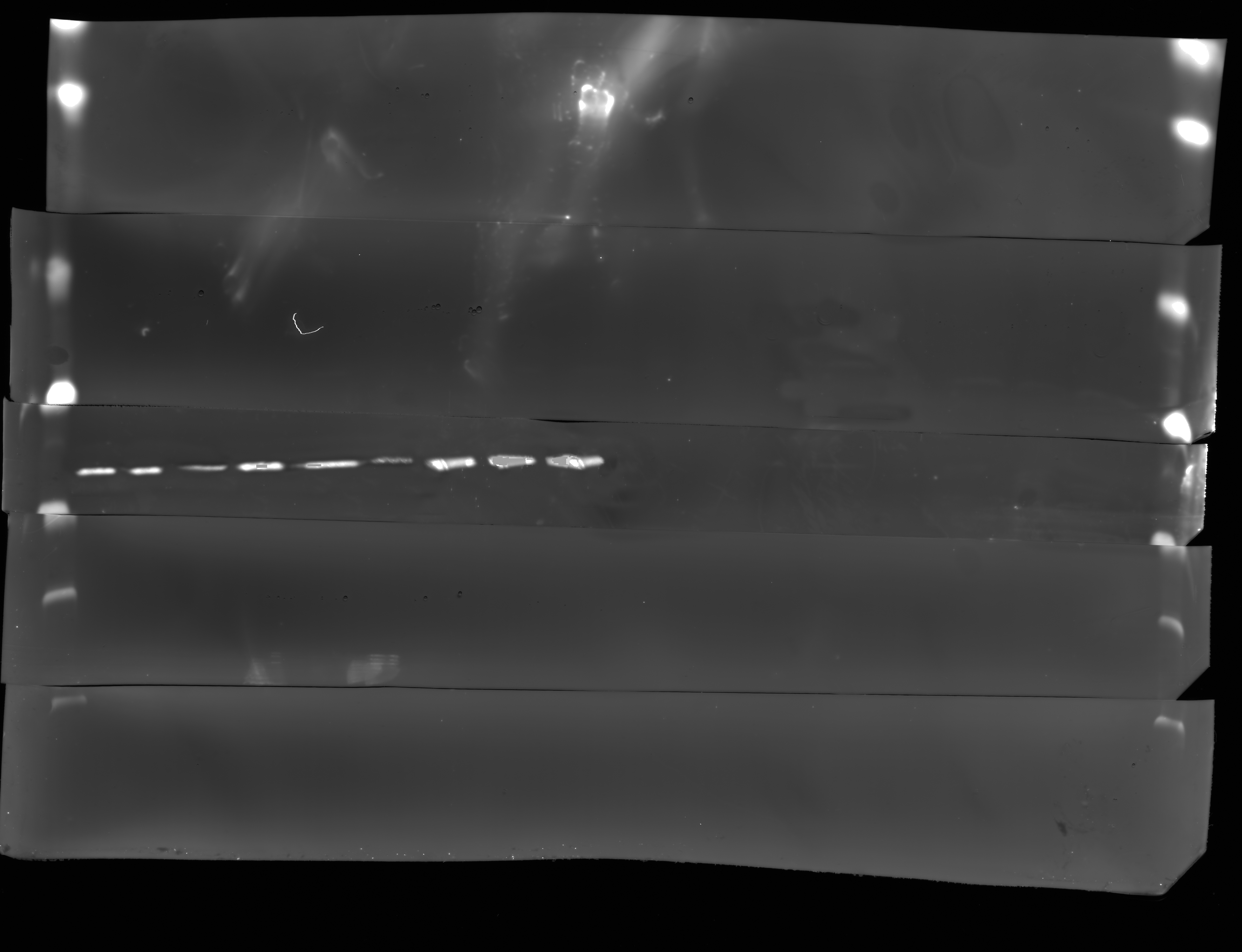

Supplement: Figure 2—figure supplement 1—source data 1. [file elife-76804-fig2-figsupp1-data1.zip › Figure 2-figure supplement 1-source data 2-exposure adjusted.TIF]

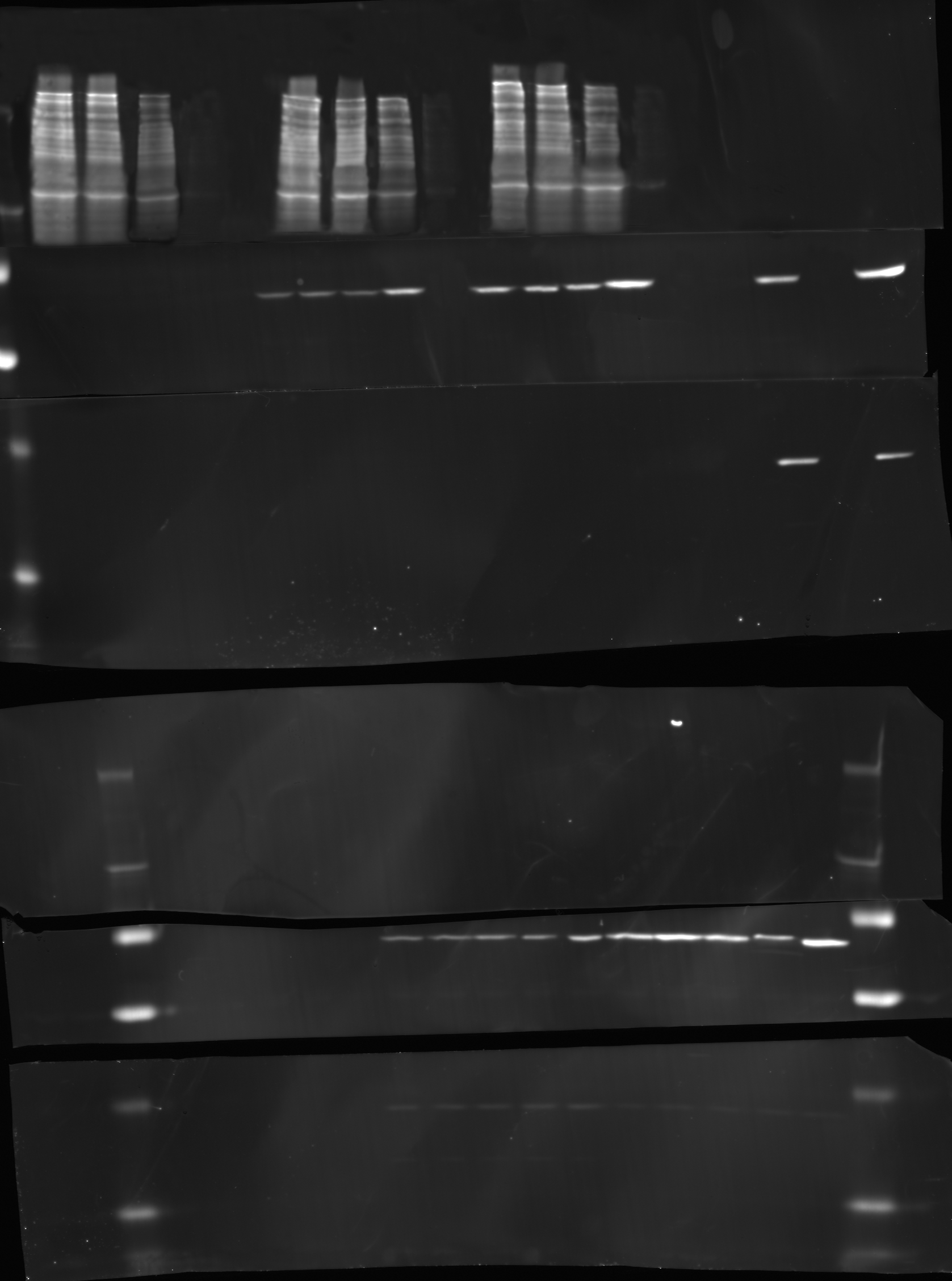

Supplement: Figure 5—source data 1. — Tegument shields MxB binding sites on HSV-1 capsids in Figure 5B. MxB requires GTP binding, but no NTE, GTP hydrolysis or dimerization to bind capsids in Figure 5C. [file elife-76804-fig5-data1.zip › Figure 5A-source data 1-exposure adjusted.tif]

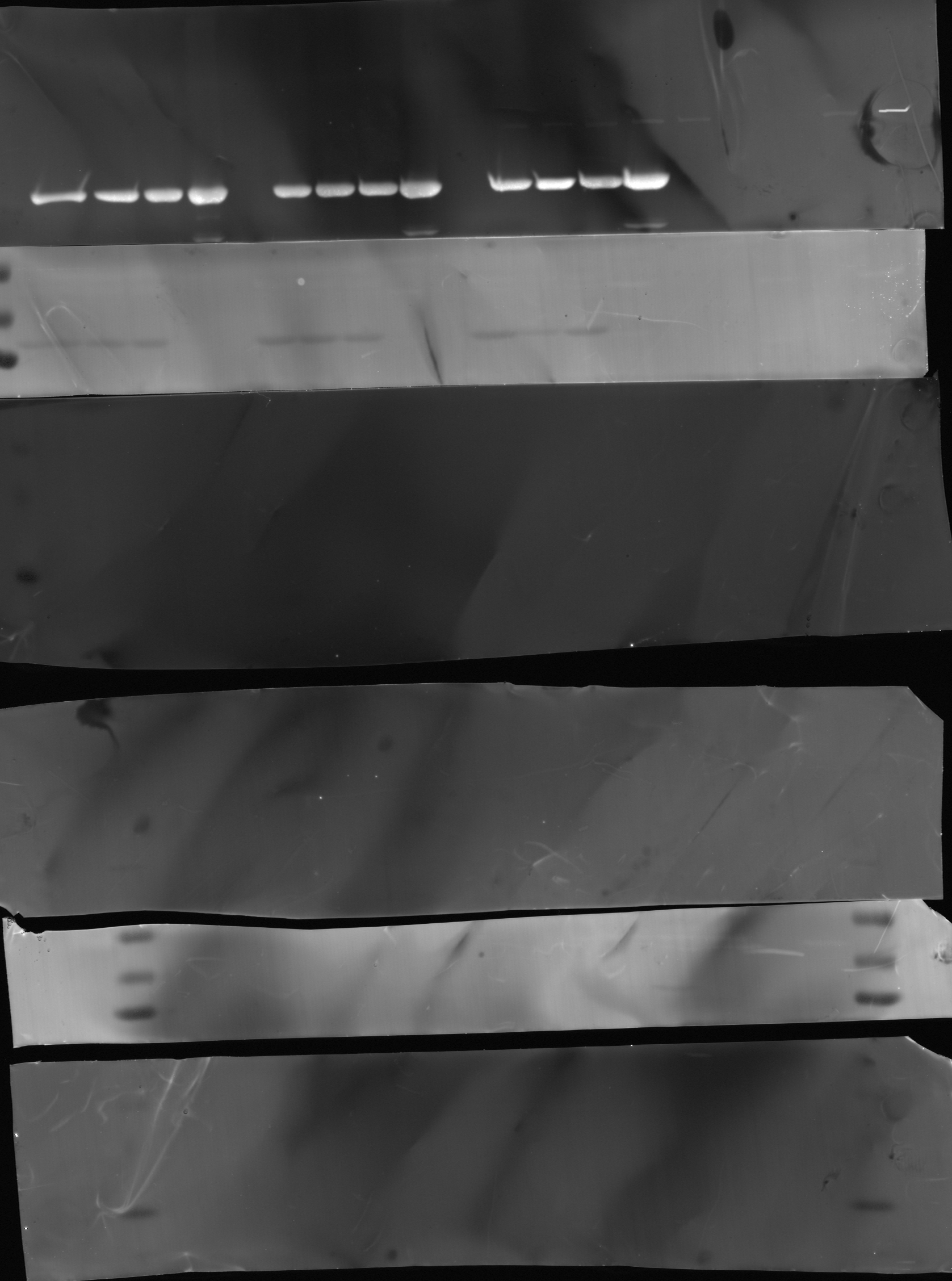

Supplement: Figure 5—source data 1. — Tegument shields MxB binding sites on HSV-1 capsids in Figure 5B. MxB requires GTP binding, but no NTE, GTP hydrolysis or dimerization to bind capsids in Figure 5C. [file elife-76804-fig5-data1.zip › Figure 5A-source data 2-exposure adjusted.tif]

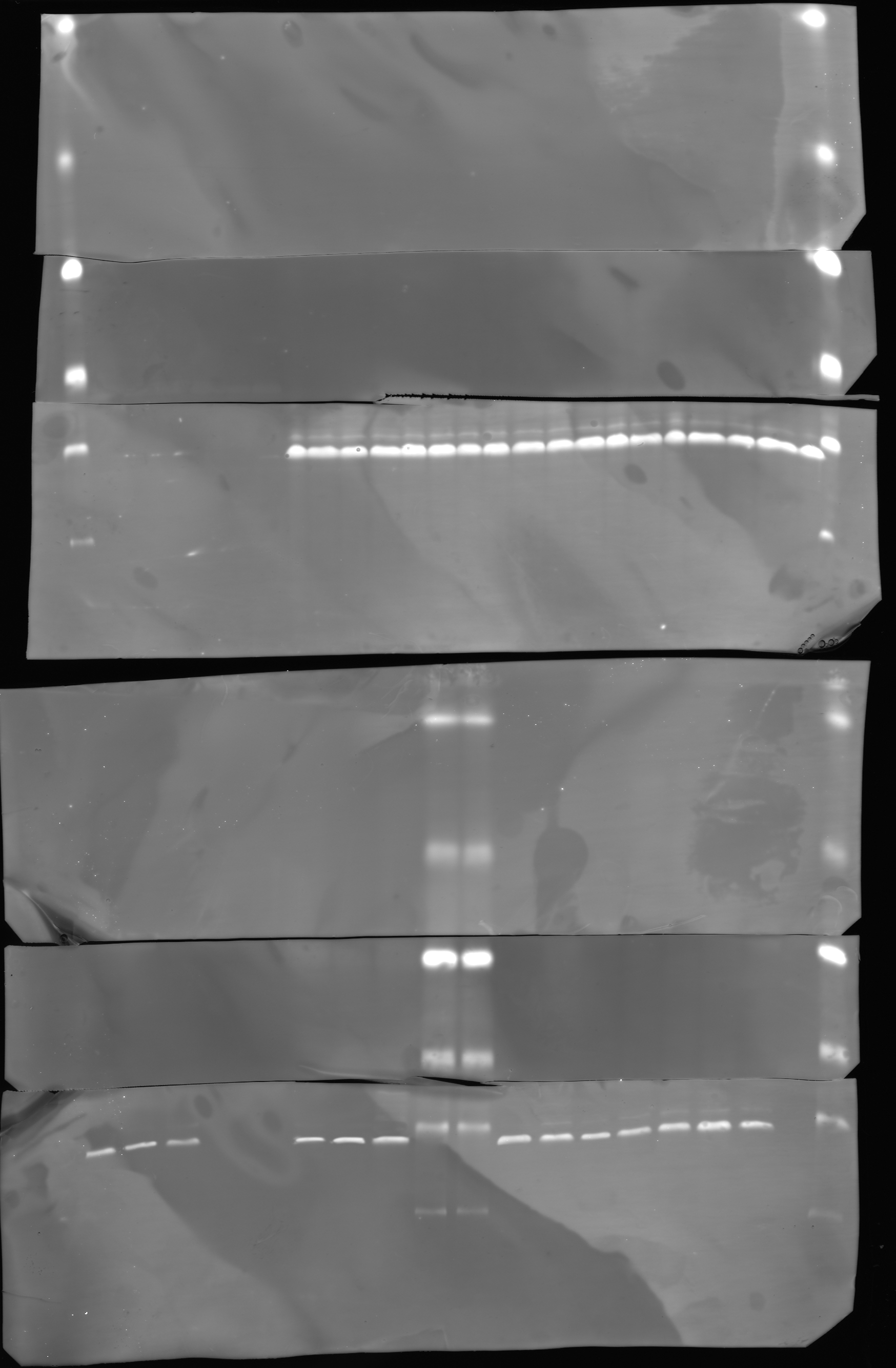

Supplement: Figure 5—source data 1. — Tegument shields MxB binding sites on HSV-1 capsids in Figure 5B. MxB requires GTP binding, but no NTE, GTP hydrolysis or dimerization to bind capsids in Figure 5C. [file elife-76804-fig5-data1.zip › Figure 5B-source data 1-exposure adjusted.tif]

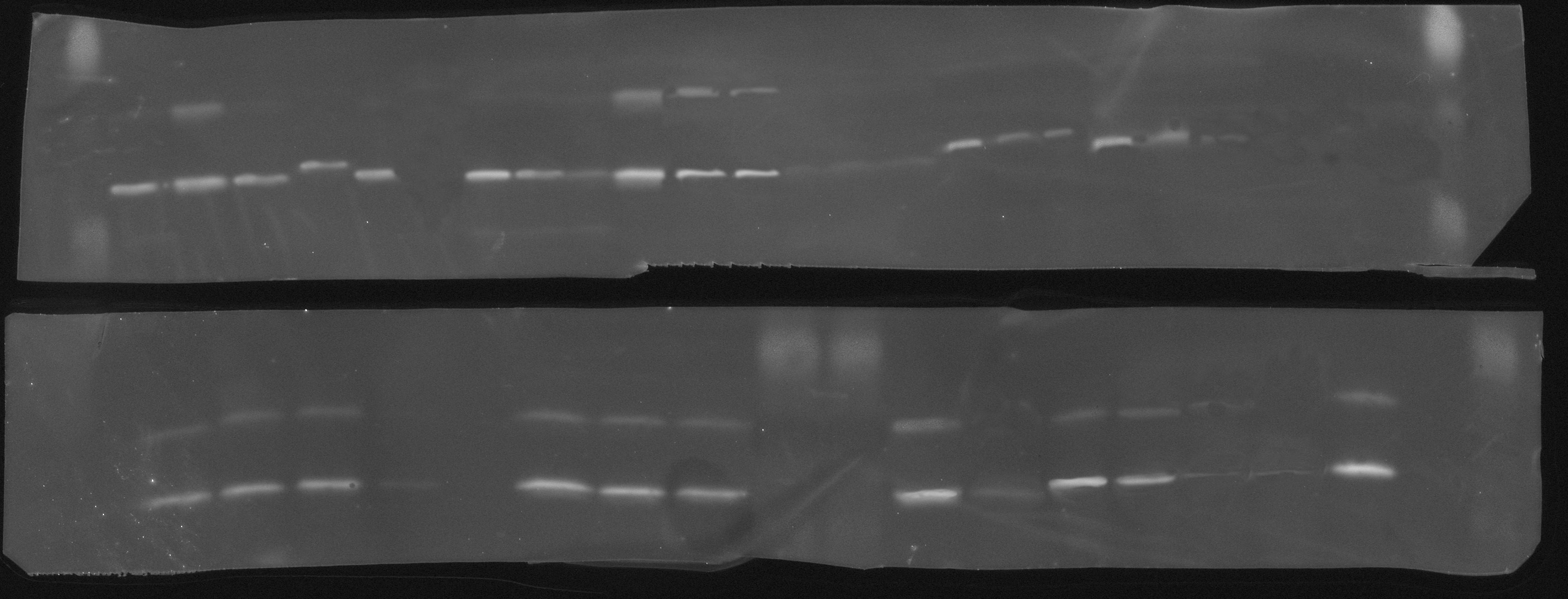

Supplement: Figure 5—source data 1. — Tegument shields MxB binding sites on HSV-1 capsids in Figure 5B. MxB requires GTP binding, but no NTE, GTP hydrolysis or dimerization to bind capsids in Figure 5C. [file elife-76804-fig5-data1.zip › Figure 5B-source data 2-exposure adjusted.tif]

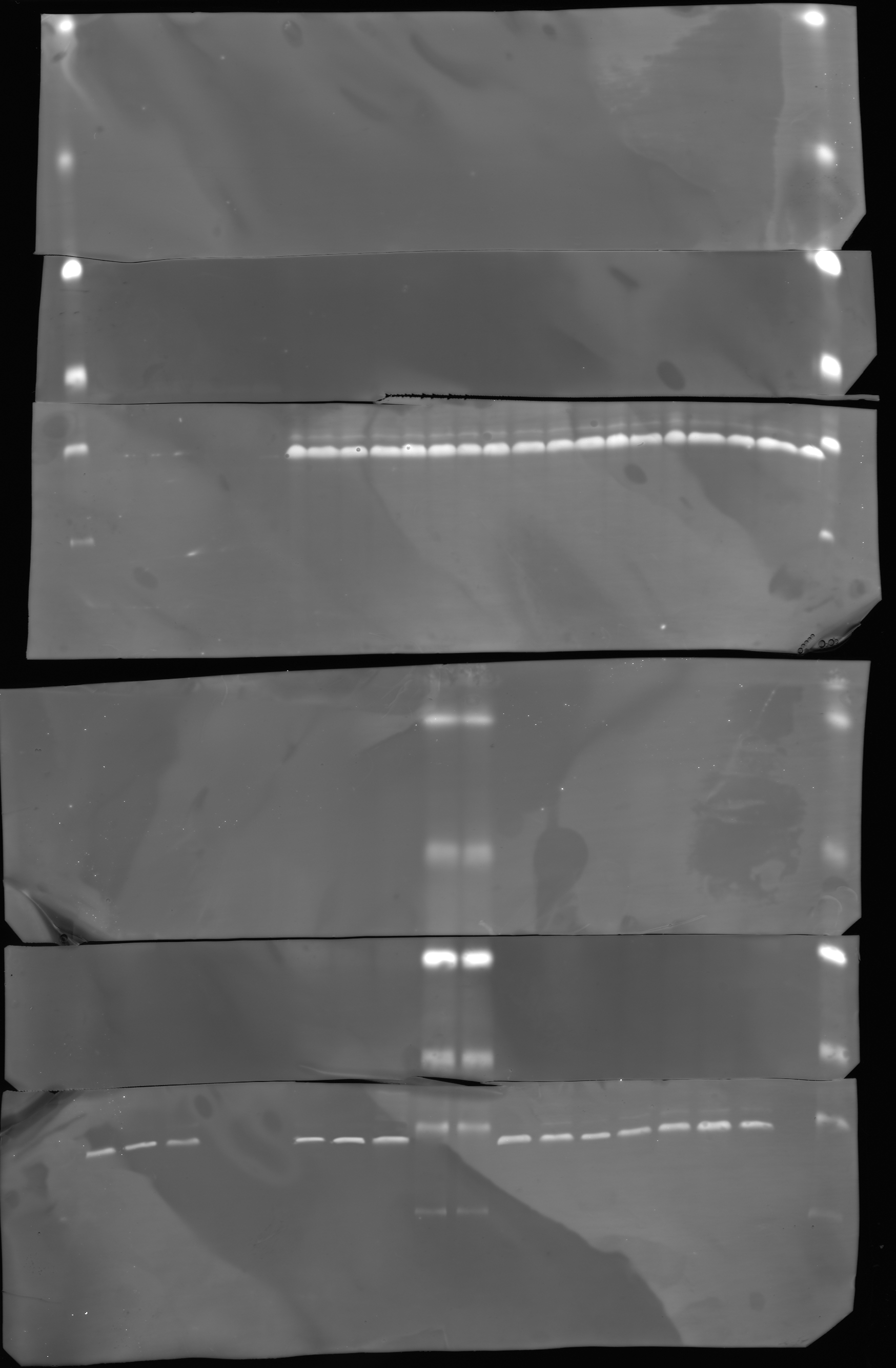

Supplement: Figure 5—source data 1. — Tegument shields MxB binding sites on HSV-1 capsids in Figure 5B. MxB requires GTP binding, but no NTE, GTP hydrolysis or dimerization to bind capsids in Figure 5C. [file elife-76804-fig5-data1.zip › Figure 5C-source data 1-exposure adjusted.tif]

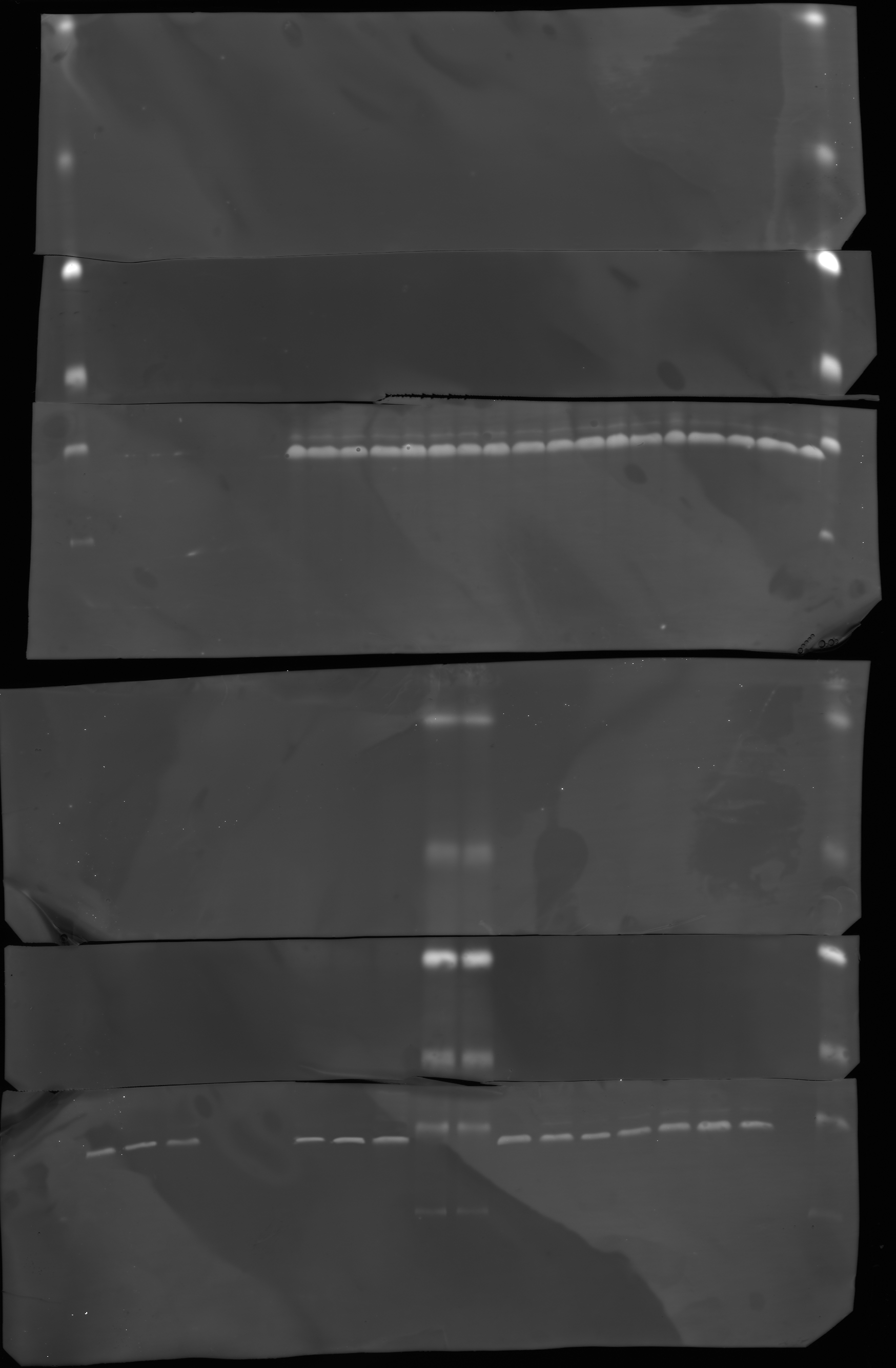

Supplement: Figure 5—source data 1. — Tegument shields MxB binding sites on HSV-1 capsids in Figure 5B. MxB requires GTP binding, but no NTE, GTP hydrolysis or dimerization to bind capsids in Figure 5C. [file elife-76804-fig5-data1.zip › Figure 5C-source data 2-exposure adjusted.tif]

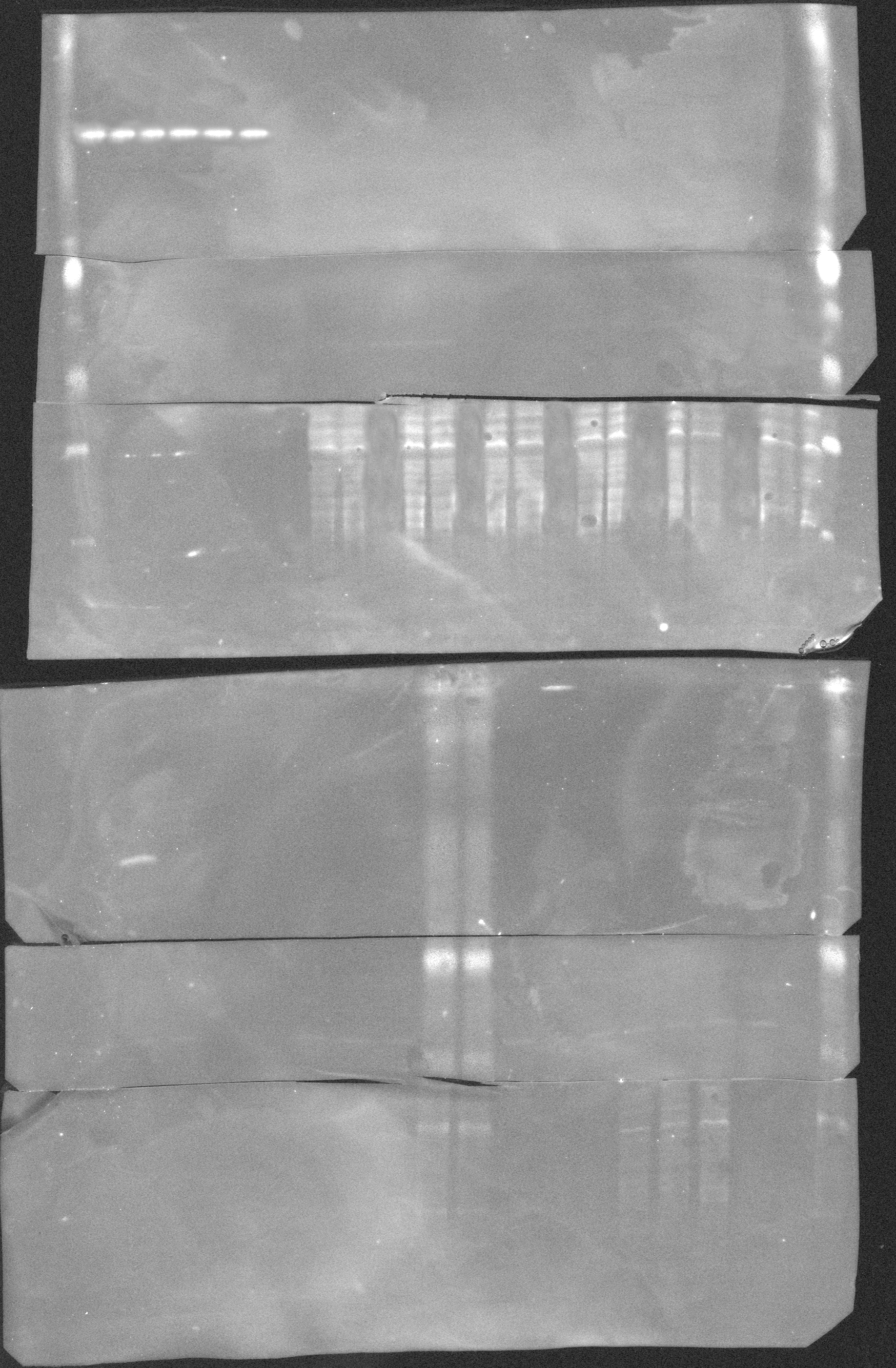

Supplement: Figure 5—source data 1. — Tegument shields MxB binding sites on HSV-1 capsids in Figure 5B. MxB requires GTP binding, but no NTE, GTP hydrolysis or dimerization to bind capsids in Figure 5C. [file elife-76804-fig5-data1.zip › Figure 5C-source data 3-exposure adjusted.tif]

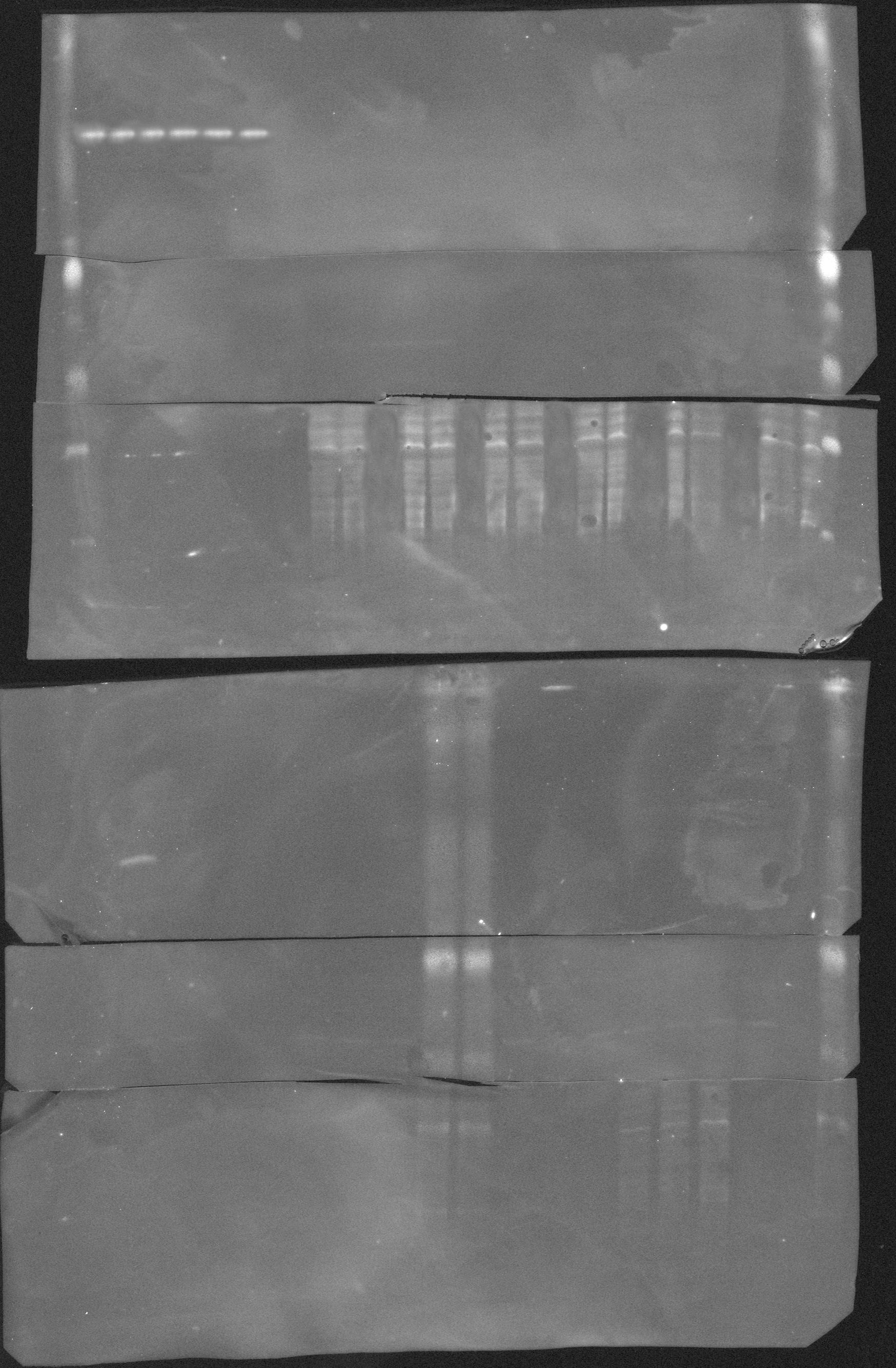

Supplement: Figure 5—source data 1. — Tegument shields MxB binding sites on HSV-1 capsids in Figure 5B. MxB requires GTP binding, but no NTE, GTP hydrolysis or dimerization to bind capsids in Figure 5C. [file elife-76804-fig5-data1.zip › Figure 5C-source data 4-exposure adjusted.tif]

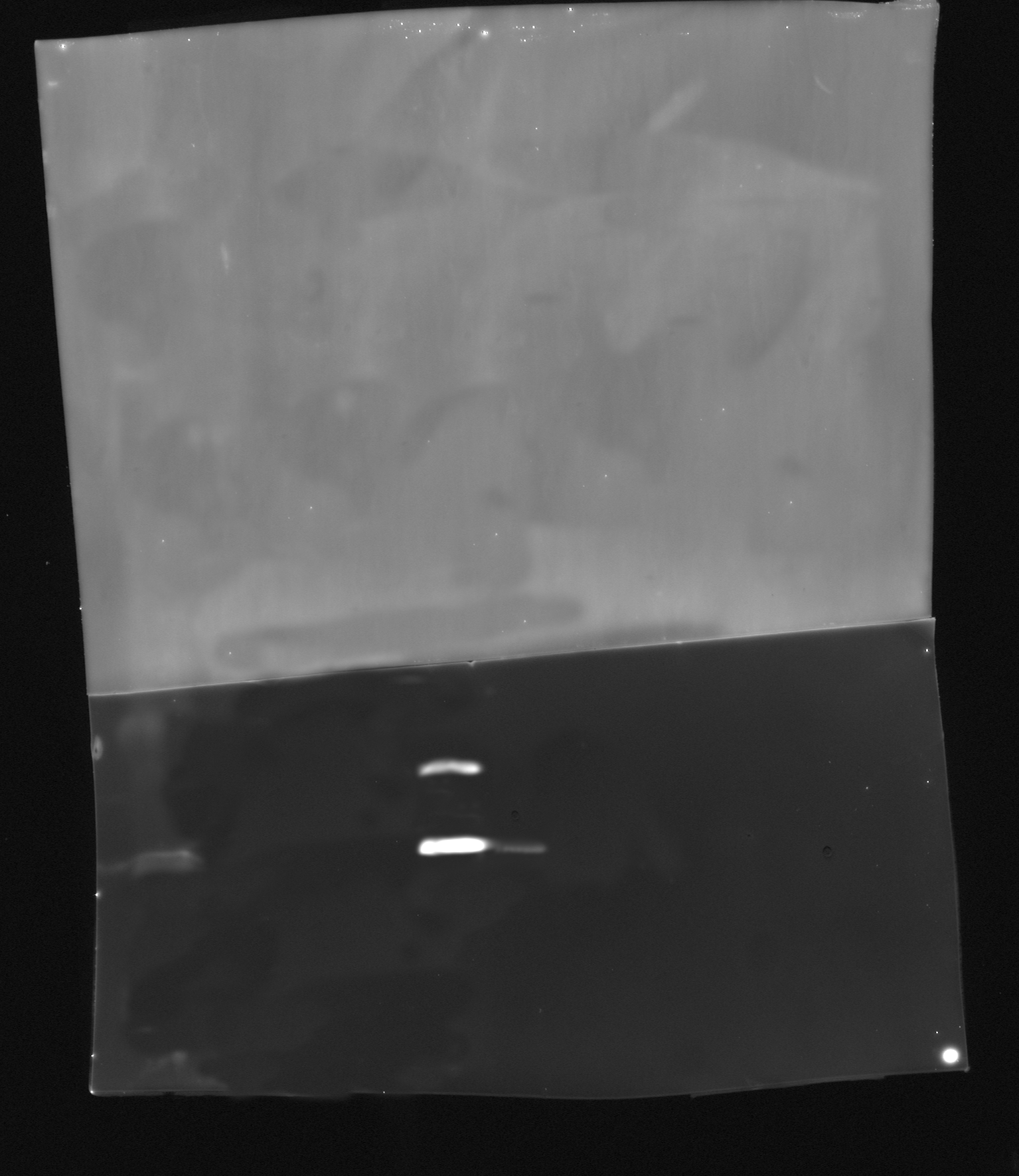

Supplement: Figure 7—figure supplement 1—source data 1. [file elife-76804-fig7-figsupp1-data1.zip › Figure 7-supplement 1-source data 1-exposure adjusted.tif]

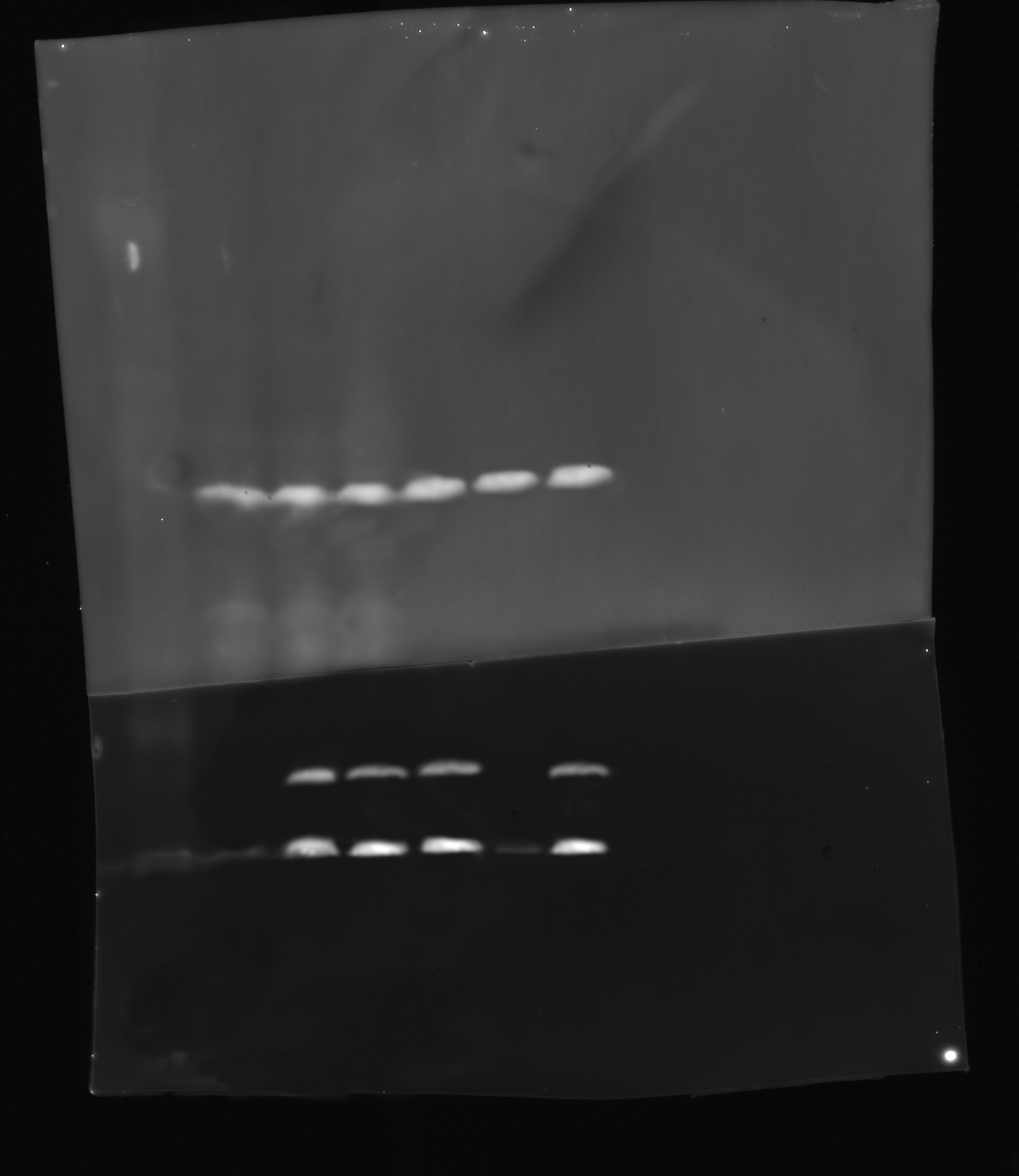

Supplement: Figure 7—figure supplement 1—source data 1. [file elife-76804-fig7-figsupp1-data1.zip › Figure 7-supplement 1-source data 2-exposure adjusted.tif]
