# Supplementary material for: The interferon-inducible GTPase MxB promotes capsid disassembly and genome release of herpesviruses
Source: eLife. 2022 Apr 27;11:e76804. doi: 10.7554/eLife.76804 (PMC9150894; doi:10.7554/eLife.76804)
Supplement: Supplementary file 4. — mAb: monoclonal antibody. pAb: polyclonal antibody.Anti-capsid SY4563 (Döhner et al., 2018); Anti-VP5 NC-1 (Cohen et al., 1980); anti-calnexin (Hammond and Helenius, 1994); Anti-MxA/MxB M143 (Flohr et al., 1999). [file elife-76804-supp4.docx]

| Antigen | Name | species, type | Source and Reference |
| --- | --- | --- | --- |
| HSV-1 proteins | | | |
| capsid | SY4563 | rabbit pAb | (Döhner *et al.*, 2018) |
| VP5 | NC-1 | rabbit mAb | Gary Cohen & Roselyn Eisenberg, University of Pennsylvania, Philadelphia, USA; (Cohen, G. H. *et al.*, 1980) |
| Host proteins | | | |
| nuclear pore | mAb414 | mouse mAb | ab24609, Abcam |
| p230 | p230 | mouse mAb | 611280, BD Biosciences |
| E-cadherin | α-E-cadherin | mouse mAb | C37020;610404, BD Transduction Laboratories |
| calnexin | α-calnexin | rabbit pAb | Ari Helenius, ETH Zürich, Switzerland; (Hammond & Helenius, 1994) |
| Tom20 | F-10 | mouse mAb | Sc-17764, Santa Cruz Biotechnology |
| GAPDH | 14C10 | rabbit pAb | 2118S, Cell Signaling (NEB) |
| MxA/MxB | M143 | mouse mAb | (Flohr *et al.*, 1999) |
| MxA | α-Mx1 | rabbit pAb | ab207414, Abcam |
| MxB | α-Mx2 | rabbit pAb | NBP1-81018, Novus Biological |
| FLAG | ANTI-FLAG | rabbit pAb | F7425, Sigma-Aldrich |
